# Identification and validation of metabolism-related hub genes in idiopathic pulmonary fibrosis

**DOI:** 10.3389/fgene.2023.1058582

**Published:** 2023-02-27

**Authors:** Youjie Zeng, Jun Huang, Ren Guo, Si Cao, Heng Yang, Wen Ouyang

**Affiliations:** ^1^ Department of Anesthesiology, Third Xiangya Hospital, Central South University, Changsha, China; ^2^ Department of Pharmacy, Third Xiangya Hospital, Central South University, Changsha, China; ^3^ Department of Neurology, Third Xiangya Hospital, Central South University, Changsha, China

**Keywords:** IPF, metabolism, metabolic, bioinformatics, hub genes, gene expression omnibus, differentially expressed genes, biomarker

## Abstract

**Background:** Idiopathic pulmonary fibrosis (IPF) is a fatal and irreversible interstitial lung disease. The specific mechanisms involved in the pathogenesis of IPF are not fully understood, while metabolic dysregulation has recently been demonstrated to contribute to IPF. This study aims to identify key metabolism-related genes involved in the progression of IPF, providing new insights into the pathogenesis of IPF.

**Methods:** We downloaded four datasets (GSE32537, GSE110147, GSE150910, and GSE92592) from the Gene Expression Omnibus (GEO) database and identified differentially expressed metabolism-related genes (DEMRGs) in lung tissues of IPF by comprehensive analysis. Then, we performed GO, KEGG, and Reactome enrichment analyses of the DEMRGs. Subsequently, key DEMRGs were identified by machine-learning algorithms. Next, miRNAs regulating these key DEMRGs were predicted by integrating the GSE32538 (IPF miRNA dataset) and the miRWalk database. The Cytoscape software was used to visualize miRNA-mRNA regulatory networks. In addition, the relative levels of immune cells were assessed by the CIBERSORT algorithm, and the correlation of key DEMRGs with immune cells was calculated. Finally, the mRNA expression of the key DEMRGs was validated in two external independent datasets and an *in vivo* experiment.

**Results:** A total of 101 DEMRGs (51 upregulated and 50 downregulated) were identified. Six key DEMRGs (ENPP3, ENTPD1, GPX3, PDE7B, PNMT, and POLR3H) were further identified using two machine-learning algorithms (LASSO and SVM-RFE). In the lung tissue of IPF patients, the expression levels of ENPP3, ENTPD1, and PDE7B were upregulated, and the expression levels of GPX3, PNMT, and POLR3H were downregulated. In addition, the miRNA-mRNA regulatory network of key DEMRGs was constructed. Then, the expression levels of key DEMRGs were validated in two independent external datasets (GSE53845 and GSE213001). Finally, we verified the key DEMRGs in the lung tissue of bleomycin-induced pulmonary fibrosis mice by qRT-PCR.

**Conclusion:** Our study identified key metabolism-related genes that are differentially expressed in the lung tissue of IPF patients. Our study emphasizes the critical role of metabolic dysregulation in IPF, offers potential therapeutic targets, and provides new insights for future studies.

## 1 Introduction

Idiopathic pulmonary fibrosis (IPF) is a progressive, life-threatening, chronic interstitial lung disease of unknown etiology (Noble et al., 2012). It is characterized by progressive scarring of the lung parenchyma, accompanied by a continuous deterioration of respiratory symptoms and a decline in lung function, ultimately leading to death (Raghu et al., 2018). Approximately two to 3 years is the median survival time for patients with IPF after diagnosis (Ley et al., 2011). There is a higher prevalence of IPF in the elderly, and the mean age of patients with IPF is around 65–70 years (Maher et al., 2021). The FDA currently approves two antifibrotic drugs (nintedanib and pirfenidone) for IPF, which only slow, not stop, fibrosis progression (Saito et al., 2019). IPF is currently curable only through lung transplantation (Shenderov et al., 2021). Despite identifying several candidate biomarkers for IPF, none of these markers have yet been translated into clinical practice (Ley et al., 2014). Thus, there is an urgent need to explore the pathophysiological mechanisms of IPF further and develop new targeted therapeutic strategies.

An increasing number of studies have recently demonstrated the role of metabolic dysregulation in IPF. For instance, Kang et al. reported altered glycolysis and glutamine metabolism in human lungs with severe IPF (Kang et al., 2016). Furthermore, proteomics studies revealed dysregulated levels of transcription factors NF-kB, PPARγ, and c-myc in bronchoalveolar lavage fluid (BALF) from IPF patients compared to healthy controls (Landi et al., 2014). Interestingly, these transcription factors have been reported to participate in numerous metabolic dysregulation mechanisms (Kauppinen et al., 2013; Jiang et al., 2017; Botta et al., 2018). In addition, lung fibroblasts and alveolar epithelial cells have been observed to display profibrotic phenotypes due to dysregulated lipid metabolism (Mamazhakypov et al., 2019). A recent review summarized the proteins dysregulated in IPF involving the renin-angiotensin-aldosterone system, hypoxia, oxidative stress, iron metabolism, dysregulated lipid metabolism, and mitochondrial alterations, highlighting the potential impact of metabolic dysregulation in IPF (Bargagli et al., 2020). Conclusively, there is an inescapable relationship between metabolic dysregulation and IPF, and the search for novel metabolism-related markers can help further understand the metabolism-related pathological molecular mechanisms of IPF. Rectifying these metabolic alterations is emerging as a promising new strategy for antifibrotic therapy.

Our study first analyzed GSE32537, GSE110147, GSE150910, and GSE92592 from the Gene Expression Omnibus (GEO) database and identified differentially expressed metabolism-related genes (DEMRGs) in the lung tissue of IPF patients. Subsequently, we conducted a functional enrichment analysis of DEMRGs. Then, we used two machine-learning methods, least absolute shrinkage and selection operator (LASSO) regression and support vector machine recursive feature elimination (SVM-RFE), to identify six IPF signature genes as key DEMRGs: ENPP3, ENTPD1, PDE7B, GPX3, PNMT, and POLR3H. The expression of ENPP3, ENTPD1, and PDE7B was significantly upregulated in IPF patients’ lung tissue, and the expression of GPX3, PNMT, and POLR3H was significantly downregulated. Afterward, we predicted miRNAs regulating key DEMRGs using the miRWalk database, combining it with the GSE32538 dataset (miRNA microarray expression profiles of IPF) to construct a miRNA-mRNA regulatory network. Next, the relative levels of immune cells were assessed by the CIBERSORT algorithm, and the correlation of key DEMRGs with immune cells was calculated. Finally, we validated the expression patterns of six key DEMRGs by analyzing the external independent dataset GSE53845 and performing qRT-PCR.

## 2 Materials and methods

### 2.1 Study design

**FIGURE 1:**
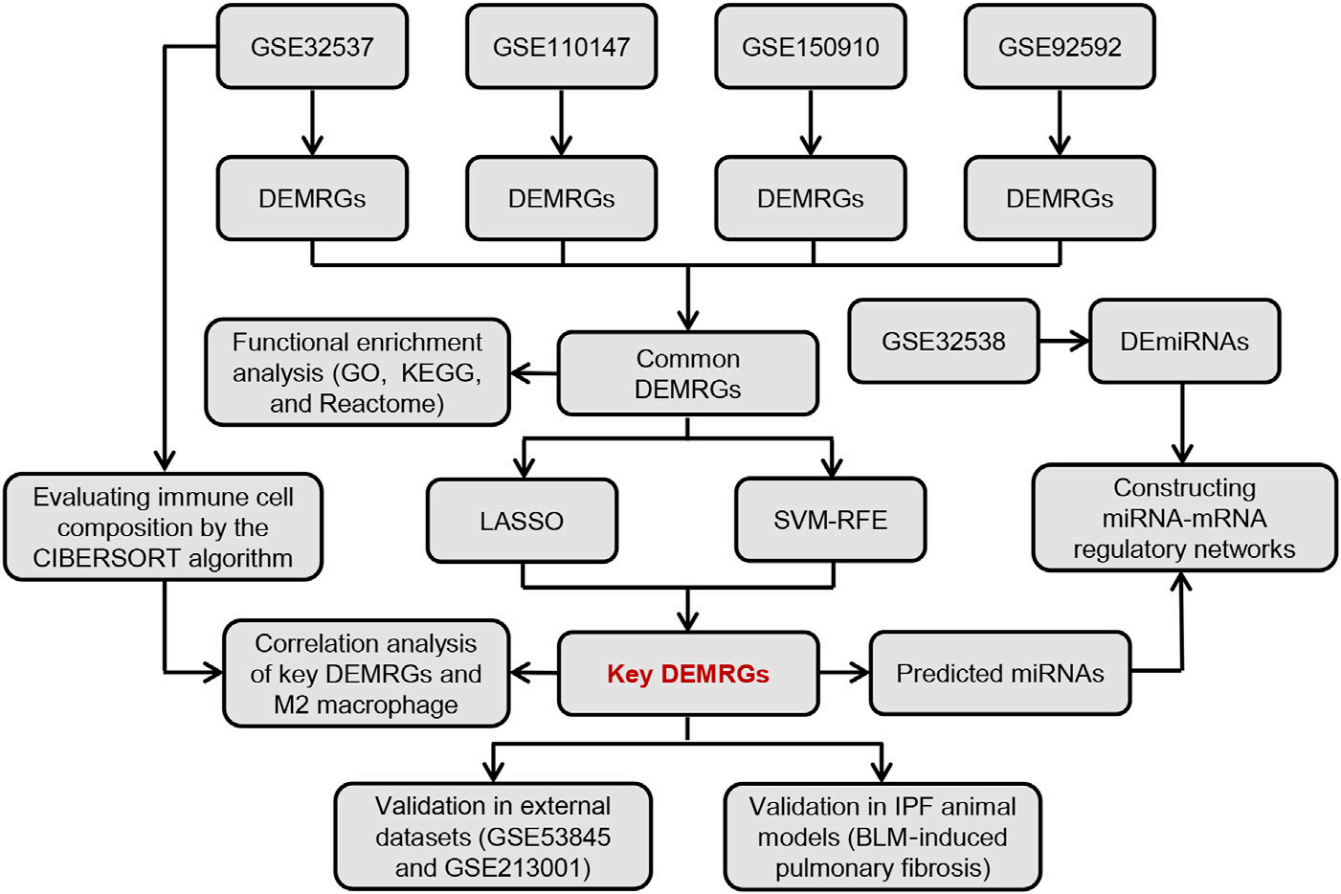
The overall flow chart of this study.

Figure 1 shows the overall flow chart of this study. First, we performed differential expression analysis on four GEO gene expression profile datasets to identify common differentially expressed metabolism-related genes (DEMRGs) in IPF lung tissues. Subsequently, we performed a functional enrichment analysis for these common DEMRGs. Then, we identified key DEMRGs using two machine-learning algorithms. Finally, we constructed potential miRNA-mRNA regulatory networks for key DEMRGs, calculated the correlation of key DEMRGs with immune cell levels, and validated the expression of key DEMRGs in external GEO datasets and animal models.

### 2.2 Gene expression profile data

Our study obtained publicly available datasets from the Gene Expression Omnibus (GEO; https://www.ncbi.nlm.nih.gov/geo/) database (Edgar et al., 2002). The GEO database is a public database that stores extensive publicly available high-throughput gene expression and other functional genomics datasets (Clough and Barrett, 2016). All datasets downloaded in this study stored gene expression at the mRNA level (array or high-throughput sequencing), and samples in the dataset were obtained from the lung tissue of IPF patients and healthy control individuals. First, we performed a comprehensive bioinformatics analysis of GSE32537, GSE110147, GSE150910, and GSE92592 to identify key DEMRGs. Then, using GSE32538, we constructed a miRNA-mRNA regulatory network of key DEMRGs. Finally, we validated the key DEMRGs in GSE53845 and GSE213001. Table 1 provides details of all the GEO datasets used in our study.

**TABLE 1:** Details of all the GEO datasets used in this study.

Accession number	Platform	Samples	Experiment type
GSE32537	GPL6244	119 IPF lung tissues vs 50 healthy lung tissues	Array
GSE110147	GPL6244	22 IPF lung tissues vs 11 healthy lung tissues	Array
GSE150910	GPL24676	103 IPF lung tissues vs 103 healthy lung tissues	High throughput sequencing
GSE92592	GPL11154	20 IPF lung tissues vs 19 healthy lung tissues	High throughput sequencing
GSE32538	GPL8786	106 IPF lung tissues vs 50 healthy lung tissues	Array (miRNA)
GSE53845	GPL6480	40 IPF lung tissues vs 8 healthy lung tissues	Array
GSE213001	GPL21290	62 IPF lung tissues vs 41 healthy lung tissues	High throughput sequencing

### 2.3 Screening of differentially expressed metabolism-related genes (DEMRGs)

The metabolism-related genes (MRGs) were obtained from the Molecular Signatures database (MSigDB, https://www.gsea-msigdb.org/gsea/msigdb) (Liberzon et al., 2011). Specifically, we first downloaded the KEGG gene set (c2. cp.kegg.v7.5.1. symbols.gmt) from the MSigDB, then searched for the keyword “metabolism” to obtain metabolism-related terms, and finally we integrated the genes within these selected metabolism-related trems, which were defined as MRGs to be used for subsequent analysis in our study. All metabolism-related terms and the genes within each term are shown in Supplementary Table S1.

GSE32537 and GSE110147 were based on the GPL6244 platform of Affymetrix Human Gene 1.0 ST Array. We used the RMA algorithm *via* the “oligo” R package for background correction and normalization of the raw data in the two datasets. Subsequently, differentially expressed genes were identified using the “limma” R package. GSE150910 and GSE92592 were RNA-seq datasets that were generated using the Illumina platform. We first downloaded their raw gene count matrix files. Then, we performed differential expression analysis on the gene expression matrix normalized by the vst function of the “Deseq2” R package. An adjusted *p*-value <0.05 was set as the threshold for identifying differentially expressed genes.

After acquiring the DEMRGs from each of the four GEO datasets, we used the Venn diagram to search for the common upregulated DEMRGs and the common downregulated DEMRGs. The “ggvenn” R package was applied to plot the Venn diagrams of common DEMRGs of the four datasets.

### 2.4 Functional enrichment analysis of DEMRGs

We performed an enrichment analysis of the common DEMRGs using the Database for Annotation, Visualization and Integrated Discovery (DAVID database, https://david.ncifcrf.gov/) (Huang da et al., 2009; Sherman et al., 2022). We performed three categories of enrichment analysis: Gene ontology (GO) enrichment analysis, Kyoto Encyclopedia of Genes and Genome (KEGG) pathway enrichment analysis, and Reactome pathway enrichment analysis. In addition, the GO enrichment analysis includes three sections: biological process (BP), cellular component (CC), and molecular function (MF). We downloaded the enrichment analysis results and defined the false discovery rate (FDR) < 0.05 as the significant enrichment threshold. In addition, we selected the top 10 most significantly enriched terms in each category and imported these results into the SangerBox platform to generate dot plots for visualization (Shen et al., 2022).

### 2.5 Screening of IPF key DEMRGs

To identify the most critical DEMRGs, we used two machine-learning algorithms: least absolute shrinkage and selection operator (LASSO) regression and support vector machine recursive feature elimination (SVM-RFE). The LASSO algorithm is a regression analysis method that minimizes regression coefficients through successive shrinkage operations to reduce the possibility of overfitting, thereby reducing redundancy and eliminating uncorrelated genes from these analyses (Friedman et al., 2010). The SVM-RFE algorithm is a method for feature selection based on SVM that defines the minimum classification error and avoids overfitting and thus is frequently used to select the optimal genes (Duan et al., 2005). The LASSO and SVM-RFE algorithms were implemented respectively by the “glmnet” package and the “e1071”package in R software. By using the two machine-learning algorithms, two sets of DEMRGs can be obtained, and the overlapping genes of these two sets of DEMRGs will be identified as the IPF key DEMRGs.

### 2.6 Construction of miRNA-mRNA regulatory networks for key DEMRGs

We intend to investigate further the miRNAs that regulate these key DEMRGs, so we first performed a differential expression analysis of GSE32538 (IPF miRNA expression profile microarray) to obtain differentially expressed miRNAs (DEmiRNAs). The significance threshold was set at an adjusted *p*-value <0.05. Since the IDs of the miRNAs in this dataset were derived from an older version of miRBase, we updated the miRNA IDs using the miEAA 2.0 database (Kern et al., 2020). Subsequently, we predicted miRNAs that interacted with key DEMRGs using the miRWalk database (http://mirwalk.umm.uni-heidelberg.de/) (Sticht et al., 2018). If a DEmiRNA was present in the predicted miRNAs from miRWalk, it would be included in the final miRNA-mRNA regulatory network. Therefore, the upregulated DEmiRNAs were then intersected with the predicted miRNAs that interact with downregulated key DEMRGs, while the downregulated DEmiRNAs were intersected with the predicted miRNAs that interact with upregulated key DEMRGs. Finally, we visualized the miRNA-mRNA regulatory network in the Cytoscape software (v 3.9.1) (Shannon et al., 2003).

### 2.7 Immune infiltration analysis

We assessed the relative content of immune cells of each sample in the GSE32537 dataset using the CIBERSORT algorithm in R software (Newman et al., 2015). The CIBERSORT algorithm calculates the relative expression of 22 immune cells based on the “LM22”matrix downloaded from the CIBERSORT portal (http://cibersort.stanford.edu/). First, we evaluated the relative expression of immune cells in all samples and plotted a histogram of immune cell content for each sample. Subsequently, we compared the content of each immune cell between IPF patients and healthy controls and plotted a boxplot for visualization. The Shapiro-Wilk test was performed to examine the normality of data, and the t-test or Mann-Whitney Wilcoxon test was used to conduct comparisons between groups based on the results of normality test (Supplementary Table S2). Finally, we calculated the correlation between 6 key DEMRGs and M2 macrophage content in 119 IPF patients. All results were visualized using the “ggplot2” R package.

### 2.8 Validation of key DEMRGs in independent external datasets

To improve the confidence of the results, we validated the expression of key DEMRGs in two independent external datasets (GSE53845 and GSE213001). We compared the mRNA expression levels of the key DEMRGs between IPF patients and control groups. We performed the Shapiro-Wilk test to check the normality of the data before making comparisons between groups. Based on the normality results (Supplementary Table S3, S4), we used the t-test or the Mann-Whitney Wilcoxon test to compare differences between groups. A *p*-value of <0.05 was considered statistically significant. Gene expression comparisons between groups were analyzed and visualized using the “ggplot2” package in R software (Wickham, 2016).

### 2.9 Construction of IPF animal models

The animal study was approved by the Laboratory Animal Welfare Ethics Committee of Central South University. Mice of the C57BL/6 strain (Adult male, 20 ± 2 g) were purchased from Hunan SJA Laboratory Animal Co., Ltd. (Hunan, China). Mice were housed in pathogen-free conditions with a 12 h dark/light cycle and were given access to food and water without restriction.

Single tracheal instillation of bleomycin (BLM) was applied to construct the pulmonary fibrosis model (Moeller et al., 2008). Mice were randomly divided into two groups: 1) Sham group (n = 6): intra-tracheal instillation of 50 µL saline alone; 2) BLM group (n = 6): intra-tracheal instillation of 50 µL saline containing BLM (5 mg/kg). Before surgery, mice were anesthetized by intraperitoneal injection of 1% sodium pentobarbital (50 mg/kg). All mice were euthanized 2 weeks after surgery, and their lung tissue was harvested.

### 2.10 Validation of key DEMRGs by qRT-PCR

Total RNA was extracted from lung tissue using TRIzol reagent (Invitrogen, Carlsbad, CA, United States), and qRT-PCR was performed using the ABI ViiA 7 real-time PCR system. GAPDH mRNA was used as an internal control for the key DEMRGs, and the relative fold differences were calculated using the 2^−ΔΔCT^ method. Triplicates of all experiments were performed. Table 2 presents the qRT-PCR primer sequences utilized in our study.

**TABLE 2:** qRT-PCR primer sequences.

Gene	Primer sequence (5'→ 3′)
ENPP3	F: CAG​CAA​CGG​TGA​AAG​CAA​AT
	R: CTG​ATG​TAG​TCC​CTG​TGG​TAA​AG
PDE7B	F: ACT​CTG​TTG​TGT​CAC​CTC​TTC
	R: GGT​TGT​GAC​CGT​GGT​AAT​CT
ENTPD1	F: AAC​TGT​CCA​CCG​AAC​TGA​TAC
	R: CCG​ATT​GTT​CGC​TTT​CCA​TTC
PNMT	F: GGG​ACG​GGT​TCT​CAT​TGA​TAT​T
	R: CTG​ACG​GTT​GAC​TTC​CAA​GAA
POLR3H	F: CCA​GGG​CCT​CTT​TCA​TGT​T
	R: CTG​CTC​TGC​CAC​CAG​TAT​TT
GPX3	F: CCT​TTT​AAG​CAG​TAT​GCA​GGC​A
	R: CAA​GCC​AAA​TGG​CCC​AAG​TT
GAPDH	F: GAG​CAT​CTC​CCT​CAC​AAT​TC
	R: GGGTGCAGCGAACTTTAT

Relative expression levels of the key DEMRGs were plotted in a barplot using the GraphPad Prism 8 software. Based on the normality results calculated by Shapiro-Wilk (Supplementary Table S5), the differences between groups were calculated using the t-test or Mann-Whitney Wilcoxon test, and *p*-values <0.05 were considered statistically significant.

## 3 Results

### 3.1 Identification of differentially expressed metabolism-related genes (DEMRGs)

**FIGURE 2:**
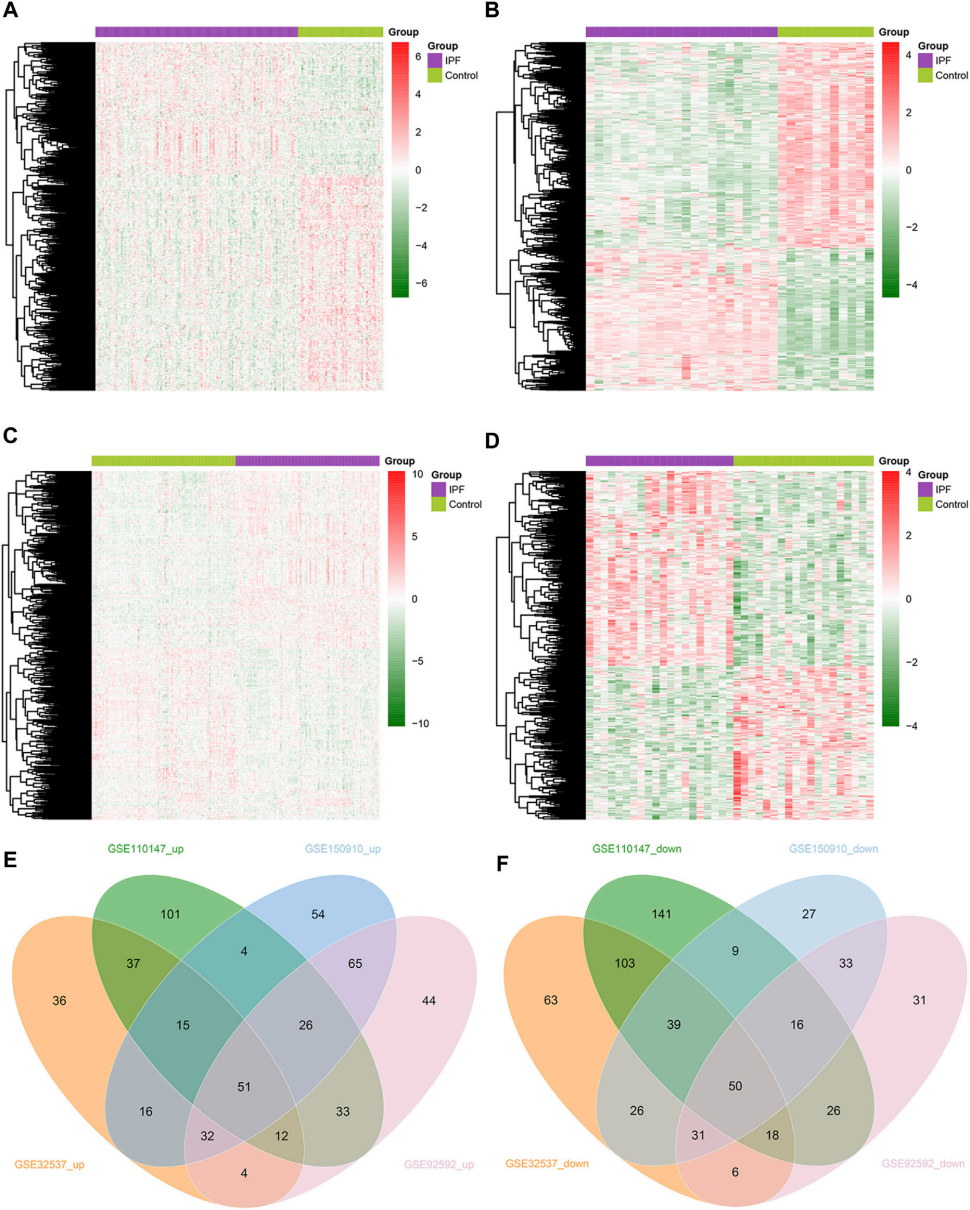
Identification of DEMRGs in IPF. **(A)** Heatmap of DEMRGs in GSE32537 (203 upregulated and 336 downregulated DEMRGs). **(B)** Heatmap of DEMRGs in GSE110147 (279 upregulated and 402 downregulated DEMRGs). **(C)** Heatmap of DEMRGs in GSE150910 (263 upregulated and 231 downregulated DEMRGs). **(D)** Heatmap of DEMRGs in GSE92592 (267 upregulated and 211 downregulated DEMRGs). **(E)** The Venn diagram identified fifty-one commonly upregulated DEMRGs. **(F)** The Venn diagram identified fifty commonly downregulated DEMRGs.

We obtained 949 unique MRGs through MSigDB. Subsequently, we performed differential expression analysis on lung tissue samples from IPF patients and healthy control individuals from four GEO datasets (GSE32537, GSE110147, GSE150910, and GSE92592), and thus obtained the differentially expressed MRGs (DEMRGs) between IPF patients and healthy controls in each dataset. As a result, in GSE32537, GSE110147, GSE150910, and GSE92592, we detected 203, 279, 263, and 267 upregulated DEMRGs, respectively. In addition, we identified 336, 402, 231, and 211 DEMRGs that were downregulated in GSE32537, GSE110147, GSE150910, and GSE92592. The heat map shows the distribution of DEMRGs in the four datasets (Figures 2A–D). The red part of the heat map indicates the upregulated DEMRGs in IPF lung tissues, while the green part indicates the downregulated DEMRGs in IPF lung tissues. The Venn diagram shows that there were 51 common upregulated DEMRGs and 50 common downregulated DEMRGs in the four datasets (Figures 2E,F). These 101 common DEMRGs were used for subsequent analysis.

### 3.2 Gene ontology, KEGG pathway, and reactome pathway enrichment analysis

**FIGURE 3:**
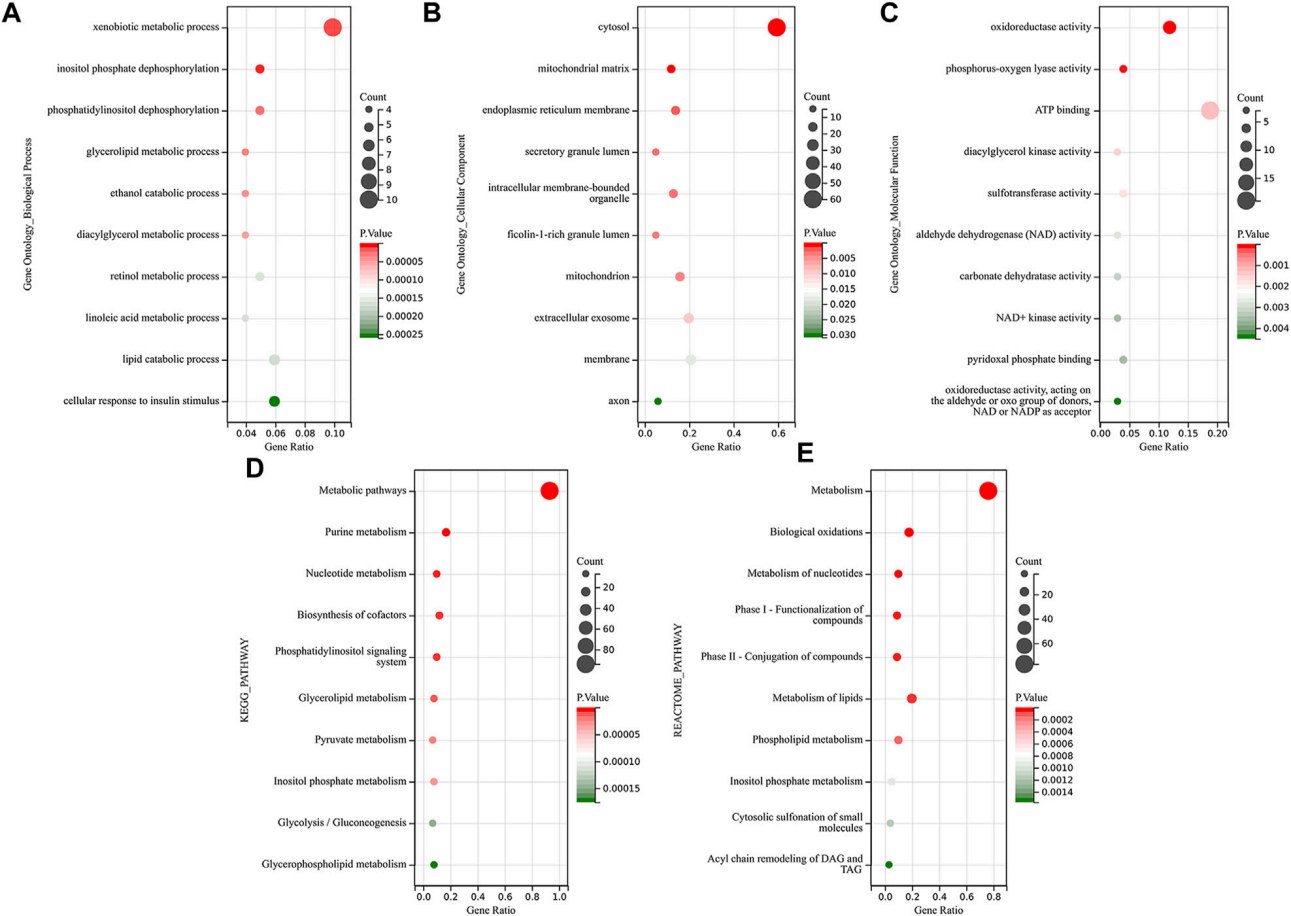
Functional enrichment analysis of DEMRGs. The dot size indicates the number of DEMRGs enriched to the corresponding term, and the dot color indicates the enrichment significance of the corresponding term. **(A)** The top 10 significantly enriched terms for Gene ontology biological process. **(B)** The top 10 significantly enriched terms for Gene ontology cellular component **(C)** The top 10 significantly enriched terms for Gene ontology molecular function. **(D)** The top 10 significantly enriched terms for the KEGG pathway. **(E)** The top 10 significantly enriched terms for the Reactome pathway.

We performed a functional enrichment analysis of these 101 common DEMRGs through the DAVID database. Figure 3 shows the top 10 significantly enriched GO, KEGG, and Reactome pathway terms. The dot size indicates the number of DEMRGs enriched to the corresponding term, and the dot color indicates the enrichment significance of the corresponding term. In the BP category of the GO enrichment analysis, DEMRGs were mainly enriched in items such as “xenobiotic metabolic process”, “inositol phosphate dephosphorylation”, and “phosphatidylinositol dephosphorylation” (Figure 3A). In the CC category of the GO enrichment analysis, these genes were mainly enriched in items such as “cytosol”, “mitochondrial matrix”, and “endoplasmic reticulum membrane” (Figure 3B). In the MF category of the GO enrichment analysis, these genes were mainly enriched in items such as “oxidoreductase activity”, “phosphorus-oxygen lyase activity”, and “ATP binding” (Figure 3C). KEGG analysis showed that DEMRGs were likely related to “metabolic pathways”, “purine metabolism”, and “nucleotide metabolism” (Figure 3D). Reactome analysis indicated that DEMRGs were significantly enriched in “metabolism”, “biological oxidations”, and “metabolism of nucleotides” (Figure 3E).

### 3.3 Identification of IPF key DEMRGs

**FIGURE 4:**
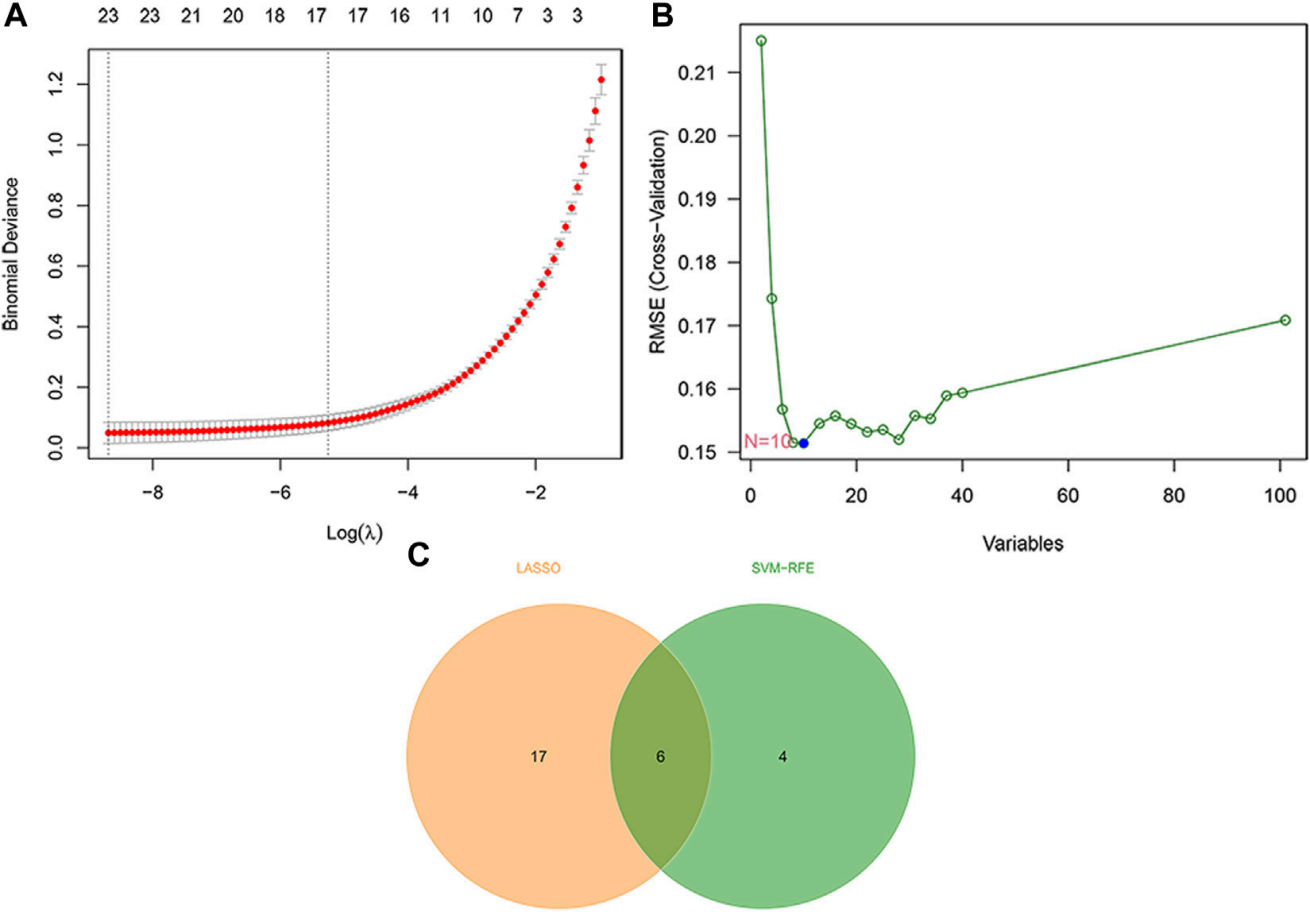
Identification of IPF key DEMRGs by using two machine-learning algorithms. **(A)** Twenty-three gene signatures were extracted *via* LASSO regression. **(B)** Ten gene signatures were extracted *via* SVM-RFE. **(C)** The Venn diagram identified six overlapping DEMRGs shared by LASSO and SVM-RFE. Therefore, the six overlapping DEMRGs were identified as key DEMRGs.

In order to identify key DEMRGs, the LASSO regression analysis was used to screen the gene signatures for the 101 common DEMRGs (Figure 4A), yielding 23 gene signatures. Furthermore, ten gene signatures were obtained using the SVM-RFE for the 101 common DEMRGs (Figure 4B). Finally, the Venn diagram showed that there were six overlapping DEMRGs (ENPP3, ENTPD1, GPX3, PDE7B, PNMT, and POLR3H) among the 23 genes identified by LASSO and the ten genes identified by SVM-RFE, and thus these six overlapping DEMRGs were defined as key DEMRGs (Figure 4C).

### 3.4 Identification of miRNA-mRNA regulatory networks of key DEMRGs

**FIGURE 5:**
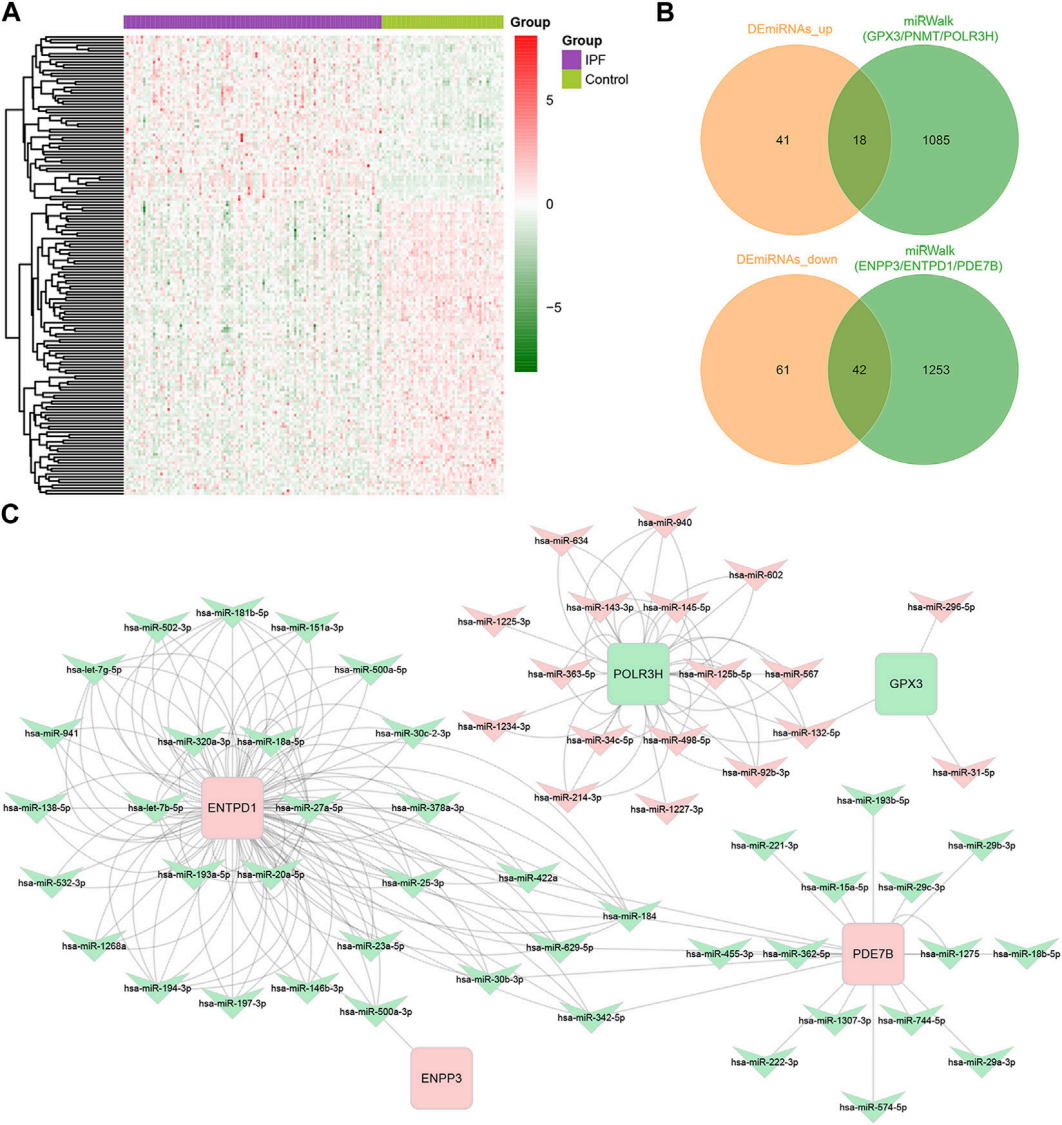
Identification of miRNA-mRNA regulatory networks of Key DEMRGs. **(A)** Heatmap of DEmiRNAs in GSE32538 (59 upregulated and 103 downregulated DEmiRNAs). **(B)** Venn diagram showing the intersecting miRNAs between DEmiRNAs and the predicted miRNAs. **(C)** The metabolism-related miRNA-mRNA regulatory network contained 60 DEmiRNAs and 5 DEMRGs. Red nodes represent upregulated key DEMRGs or DEmiRNAs in IPF lung tissue, and green nodes represent downregulated key DEMRGs or DEmiRNAs in IPF lung tissue.

After updating the miRNA IDs by the miEAA 2.0 database, we identified 59 upregulated miRNAs and 103 downregulated miRNAs in IPF from the GSE32538 dataset (Figure 5A). Using the miRWalk database, we identified 1,295 miRNAs predicted to interact with the upregulated key DEMRGs (ENPP3, ENTPD1, and PDE7B), and they had 42 overlapping miRNAs with the 103 downregulated DEmiRNAs (Figure 5B). In addition, 1,103 miRNAs predicted to interact with the downregulated key DEMRGs (GPX3, PNMT, and POLR3H) were identified through the miRWalk database, and they had 18 overlapping miRNAs with the 59 upregulated DEmiRNAs (Figure 5B). Except for PNMT, all other key DEMRGs have interactions with one or more overlapping miRNAs. Ultimately, a miRNA-mRNA regulatory network of 60 DEmiRNAs and 5 DEMRGs was constructed by Cytoscape software, with red representing upregulation and green representing downregulation (Figure 5C).

### 3.5 Immune infiltration features of IPF

**FIGURE 6:**
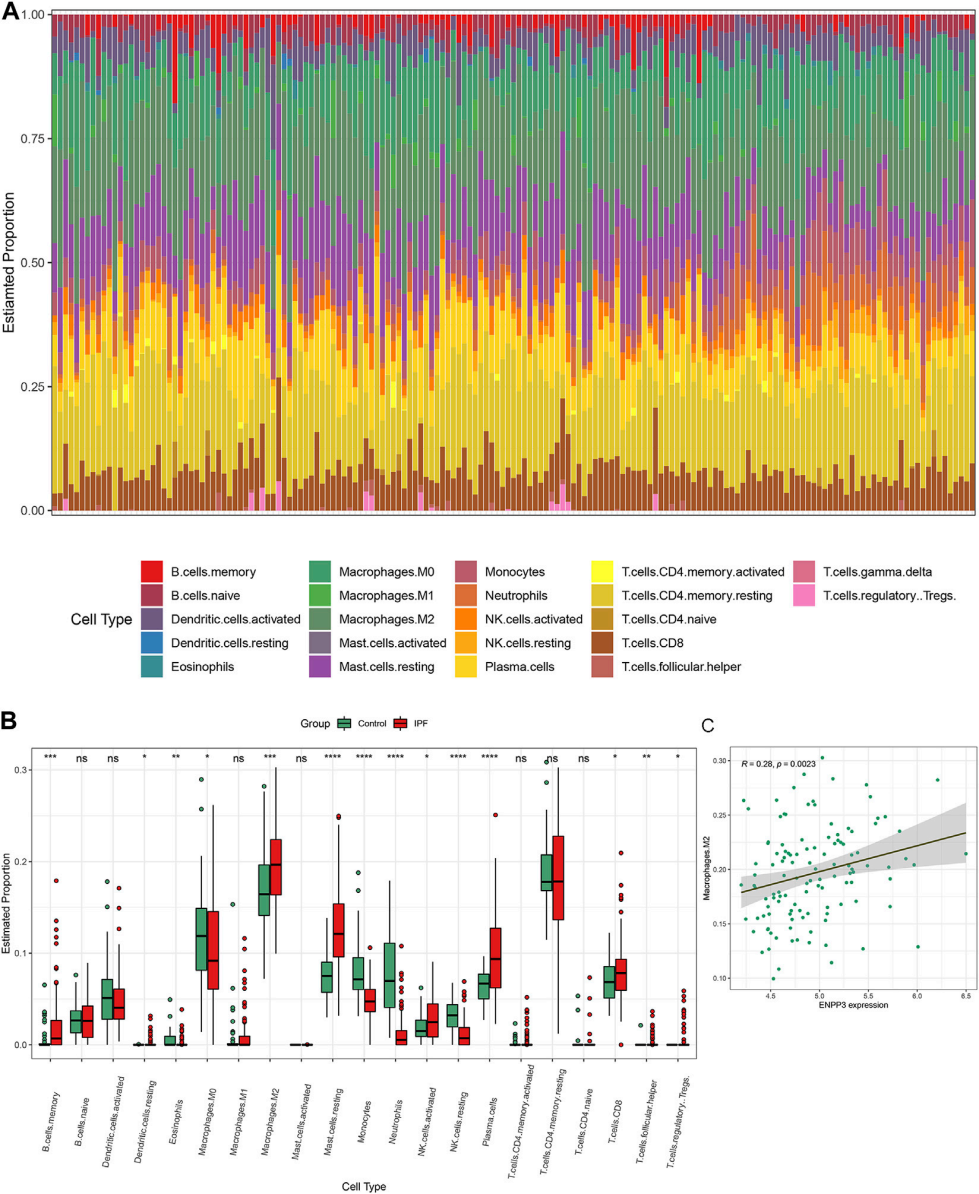
Immune cell infiltration in IPF. **(A)** Histogram of the proportion of each type of immune cell in the lung tissue of 119 IPF patients and 50 controls in the GSE32537 dataset. **(B)** Boxplot of the relative expression of each immune cell subtype between the IPF patients and healthy controls. **(C)** M2 macrophage expression was positively correlated with the expression level of ENPP3. *p < 0.05; **p < 0.01; ***p < 0.001; ****p < 0.0001.

Figure 6A presents the distribution of immune cells in the lung tissue of 119 IPF patients and 50 healthy controls in the GSE32537 dataset. The relative levels of many immune cells differed significantly between IPF and controls (Figure 6B). We focused on M2 macrophages because they contribute to the fibrotic phenotype exacerbation (Wynn and Vannella, 2016). A significant increase in M2 macrophages was found in the lung tissue of patients with IPF (Figure 6B). In addition, we calculated the correlation between the expression levels of six key DEMRGs and the expression levels of M2 macrophages. To minimize the false positive rate, correlation analysis was conducted on only 119 IPF patients. Figure 6C indicates that M2 macrophage expression was positively correlated with the expression level of ENPP3 (R = 0.28, *p* = 0.0023). Therefore, ENPP3 might be potentially associated with increased levels of M2 macrophages in the IPF process.

### 3.6 Key DEMRGs exhibited the same expression pattern in the external datasets

**FIGURE 7:**
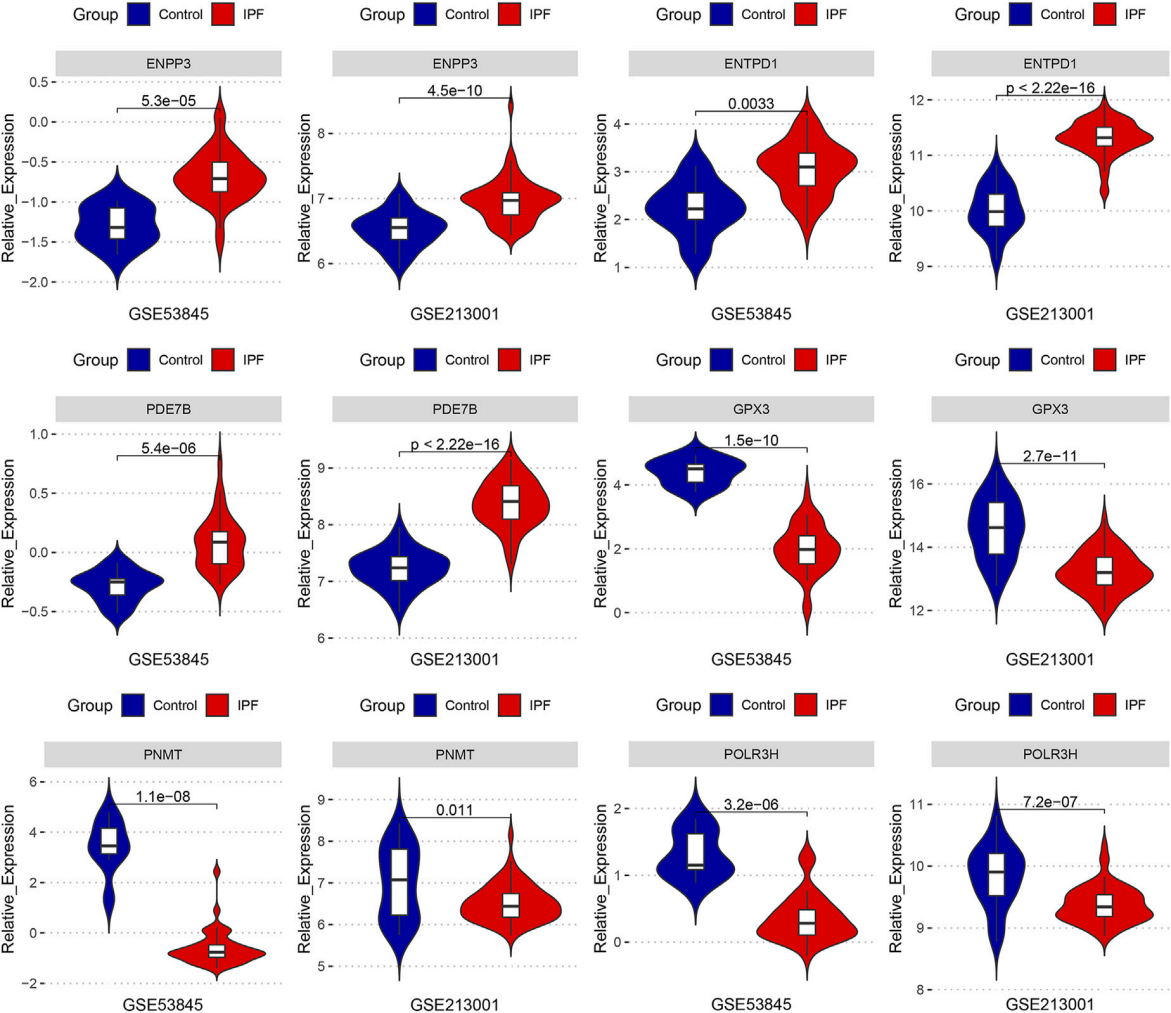
The expression levels of six key DEMRGs were validated in two independent external datasets (GSE53845 and GSE213001): ENPP3, ENTPD1, and PDE7B were significantly upregulated in IPF lung tissue (*p* < 0.05), while GPX3, PNMT, and POLR3H were significantly downregulated in IPF lung tissue (*p* < 0.05).

We compared the expression levels of six key DEMRGs in IPF patients and controls in two independent external datasets (GSE53845 and GSE213001). According to the results, ENPP3, ENTPD1, and PDE7B were significantly upregulated in the lung tissues of IPF patients (Figure 7), while GPX3, PNMT, and POLR3H were significantly downregulated (Figure 7). These results were in accordance with those in the previous four datasets.

### 3.7 Validation of the key DEMRGs by qRT-PCR

**FIGURE 8:**
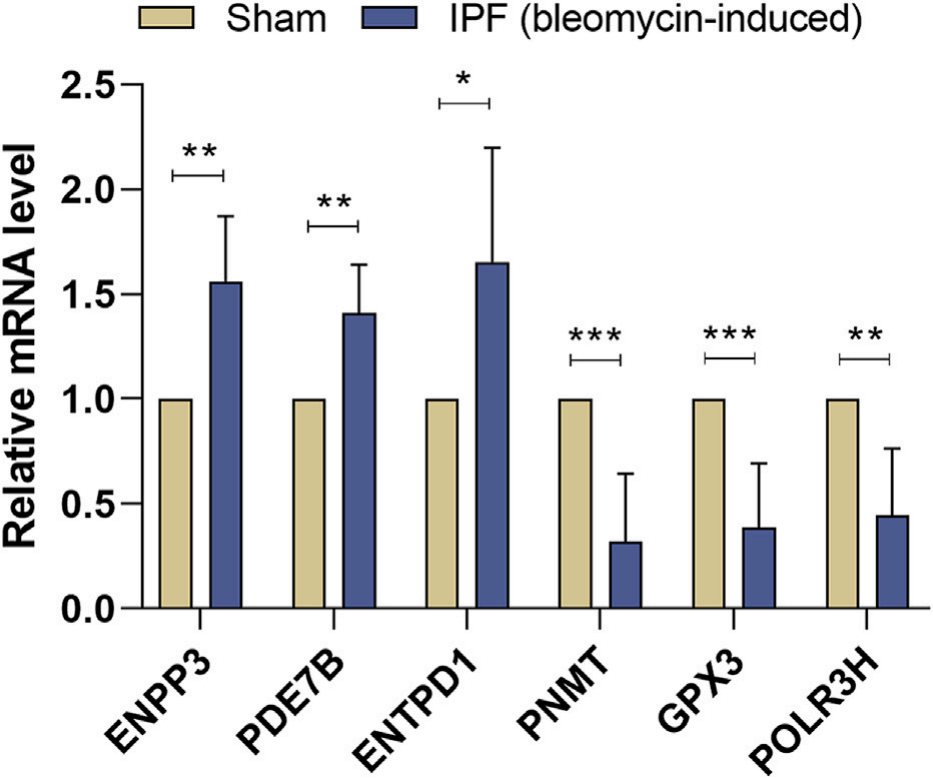
Validation of Key DEMRGs by qRT-PCR. Values represent means ± SD, n = 6/group. *p < 0.05; **p < 0.01; ***p < 0.001.

According to the results of qRT-PCR, the expression levels of ENPP3, PDE7B, and ENTPD1 were elevated, while the expression levels of PNMT, GPX3, and POLR3H were decreased in the lung tissues of bleomycin-induced pulmonary fibrosis mice compared with the sham group (Figure 8). The results of qRT-PCR remained consistent with the bioinformatics analysis; therefore, these key DEMRGs may play an essential role in the progression of IPF.

## 4 Discussion

Our study aims to identify key metabolism-related genes of IPF. First, we performed the differential analysis of the four GEO public datasets (GSE32537, GSE110147, GSE150910, and GSE92592) and integrated metabolism-related genes from the MSigDB dataset, resulting in 51 DEMRGs that were commonly upregulated and 50 DEMRGs that were commonly downregulated in the four IPF datasets. Subsequently, we performed functional enrichment analysis on these 101 DEMRGs, and the results indicated that these genes were involved in various metabolism-related terms. Then, two machine-learning algorithms were utilized to screen the key DEMRGs, resulting in six genes (ENPP3, ENTPD1, PDE7B, GPX3, PNMT, and POLR3H) as key DEMRGs. We further combined the miRNA expression profile dataset of IPF and the predicting results of the miRWalk database to construct the miRNA-mRNA network regulating the key DEMRGs. Next, we performed an immune infiltration analysis and identified an elevated M2 macrophage level in IPF patients, which reflects the enhanced M2 polarization-mediated fibrosis phenotype. In addition, the mRNA expression of the key DEMRGs was validated in two external independent datasets (GSE53845 and GSE213001). Finally, the gene expression pattern was validated by qRT-PCR, demonstrating that the key DEMRGs might have potentially significant roles in IPF.

The immune cell infiltration results showed increased levels of M2 macrophages in the lung tissue of IPF patients. As the most abundant immune cells in the lung (approximately 70%), macrophages play a critical role in pulmonary fibrosis-related airway remodeling (Cai et al., 2014). Activated macrophages are usually divided into two categories, M1 macrophages (pro-inflammatory) and M2 macrophages (anti-inflammatory/pro-fibrotic) (Vasse et al., 2021). The ENPP3 and ENTPD1 encoded products can hydrolyze ATP. Thus the elevated levels of ENPP3 and ENTPD1 observed in our study lead to a decreased ATP level. Extracellular ATP increases the global inflammation level (Cauwels et al., 2014). Besides, we identified that the M2 macrophage expression was positively correlated with the expression level of ENPP3. Taken together, we have reason to believe that ENPP3 and ENTPD1 may play a role in macrophages. The enhanced macrophage M2 polarization might be partly through the upregulation of ENPP3 and ENTPD1, leading to a decrease in ATP levels, which produces an anti-inflammatory and pro-fibrotic phenotype and ultimately exacerbates IPF. However, the specific mechanism needs to be validated in further studies.

GPX3 encodes glutathione peroxidase 3, which is expressed mainly in the lung and kidney (Lubos et al., 2011). Recent studies have shown a strong link between reactive oxygen species and fibrosis (Richter and Kietzmann, 2016). NADPH oxidase 4-derived ROS has been reported to regulate TGF-beta1-induced myofibroblast differentiation, extracellular matrix production, and contractility. A recent study uncovered a therapeutic effect of ROS-responsive liposomes in IPF, further suggesting the significance of anti-oxidative stress in IPF treatment (Liu et al., 2022). Our study shows that GPX3 expression levels are decreased in IPF lung tissue, which leads to increased levels of oxidative stress and thus exacerbates the fibrotic phenotype. Therefore, GPX3 is expected to be a potential novel target for the anti-oxidative stress treatment of IPF.

PDE7B encodes a phosphodiesterase that hydrolyzes cAMP and downregulates its signaling effects (Sasaki et al., 2000). In addition, the products of PNMT increase adrenaline production, and activation of adrenoceptors increases cAMP synthesis (Torphy, 1994; Martin et al., 2001). The decrease in cAMP results in a reduction of PKA activity and an increase in PFK activity, leading to increased F2,6BP levels. In response to the rise in F2,6BP levels, gluconeogenesis is suppressed, and glycolysis is stimulated (Pernicova and Korbonits, 2014). The increasing cellular cAMP level inhibits pulmonary fibroblast proliferation and collagen synthesis (Liu et al., 2004). In addition, glycolysis is increased early and sustainably during myofibroblast differentiation (Xie et al., 2015). The glucose transporter protein 1-dependent glycolytic phenotype was significantly increased in the lungs of aged mice, which was essential for pulmonary fibrosis (Cho et al., 2017). Actually, β-adrenergic agonists/cAMP play a key role in IPF, and β-adrenergic receptor agonists/cAMP have been shown to have beneficial effects on alveolar injury, including protection from epithelial and endothelial cell damage, restoration of alveolar fluid clearance, and reduction of fibrotic remodeling (Sriram et al., 2021). Overall, the upregulation of PDE7B and downregulation of PNMT in the lung tissues of IPF patients identified in our study might conjointly result in decreased β2-AR agonist/cAMP levels, decreased PKA activity, and enhanced glycolysis, which induced excessive collagen production and fibrosis formation.

The advantage of this study is that we have identified key metabolism-related genes that are commonly differentially expressed in IPF lung tissue using multiple bioinformatics approaches and validation in animal models. These genes may be a potential focus for future research on IPF metabolic disorders. However, several shortcomings of our study need to be acknowledged. First, the general profile of the IPF population cohort and the healthy control population cohort in the original dataset was not identical; for example, the mean age of the case group in the original study of GSE32537 was 62.6 years, whereas the mean age of the control group was 47.5 years. Therefore, it is unclear whether these differential gene expressions could be influenced by age. Nevertheless, our findings were obtained based on the analysis and validation of multiple datasets, thus minimizing the effect of potential confounding factors. The second limitation of this study is that although the identified key DEMRGs are commonly differentially expressed in IPF lung tissues, the specific degree of their impact on IPF needs to be clarified. Therefore, it will be important to interpret the findings with caution until they are validated by functional experimental research, despite the fact that they were based on reliable bioinformatics data.

## 5 Conclusion

Overall, through a comprehensive analysis of public datasets and experimental validation, we identified key metabolism-related genes that are differentially expressed in the lung tissue of IPF patients. Our study emphasizes the critical role of metabolic dysregulation in IPF, offers potential therapeutic targets, and provides new insights for future studies.

## Data Availability

Publicly available datasets were analyzed in this study. This data can be found here: Gene Expression Omnibus (GEO) database (https://www.ncbi.nlm.nih.gov/geo/; accession number: GSE32537, GSE110147, GSE150910, GSE92592, GSE32538, GSE53845, GSE213001).

## References

[B1] Bargagli E. Refini R. M. D'Alessandro M. Bergantini L. Cameli P. Vantaggiato L. (2020). Metabolic dysregulation in idiopathic pulmonary fibrosis. Int. J. Mol. Sci. 21, 5663. 10.3390/ijms21165663 32784632 PMC7461042

[B2] Botta M. Audano M. Sahebkar A. Sirtori C. R. Mitro N. Ruscica M. 2018. PPAR agonists and metabolic syndrome: An established role? Int. J. Mol. Sci., 19, 1197, 10.3390/ijms19041197 29662003 PMC5979533

[B3] Cai Y. Sugimoto C. Arainga M. Alvarez X. Didier E. S. Kuroda M. J. 2014. *In vivo* characterization of alveolar and interstitial lung macrophages in rhesus macaques: Implications for understanding lung disease in humans. J. Immunol, 192, 2821, 10.4049/jimmunol.1302269 24534529 PMC3959879

[B4] Cauwels A. Rogge E. Vandendriessche B. Shiva S. Brouckaert P. (2014). Extracellular ATP drives systemic inflammation, tissue damage and mortality. Cell Death Dis. 5, e1102. 10.1038/cddis.2014.70 24603330 PMC3973196

[B5] Cho S. J. Moon J. S. Lee C. M. Choi A. M. Stout-Delgado H. W. 2017. Glucose transporter 1-dependent glycolysis is increased during aging-related lung fibrosis, and phloretin inhibits lung fibrosis. Am. J. Respir. Cell Mol. Biol., 56, 521, 10.1165/rcmb.2016-0225OC 27997810 PMC5449513

[B6] Clough E. Barrett T. 2016. The gene expression omnibus database. Methods Mol. Biol., 1418, 93, 10.1007/978-1-4939-3578-9_5 27008011 PMC4944384

[B7] Duan K. B. Rajapakse J. C. Wang H. Azuaje F. (2005). Multiple SVM-RFE for gene selection in cancer classification with expression data. IEEE Trans. Nanobioscience 4, 228–234. 10.1109/tnb.2005.853657 16220686

[B8] Edgar R. Domrachev M. Lash A. E. (2002). Gene expression omnibus: NCBI gene expression and hybridization array data repository. Nucleic Acids Res. 30, 207–210. 10.1093/nar/30.1.207 11752295 PMC99122

[B9] Friedman J. Hastie T. Tibshirani R. 2010. Regularization paths for generalized linear models via coordinate descent. J. Stat. Softw., 33, 1, 10.18637/jss.v033.i01 20808728 PMC2929880

[B10] Huang Da W. Sherman B. T. Lempicki R. A. (2009). Systematic and integrative analysis of large gene lists using DAVID bioinformatics resources. Nat. Protoc. 4, 44–57. 10.1038/nprot.2008.211 19131956

[B11] Jiang Y. Mao C. Yang R. Yan B. Shi Y. Liu X. (2017). EGLN1/c-Myc induced lymphoid-specific helicase inhibits ferroptosis through lipid metabolic gene expression changes. Theranostics 7, 3293–3305. 10.7150/thno.19988 28900510 PMC5595132

[B12] Kang Y. P. Lee S. B. Lee J. M. Kim H. M. Hong J. Y. Lee W. J. (2016). Metabolic profiling regarding pathogenesis of idiopathic pulmonary fibrosis. J. Proteome Res. 15, 1717–1724. 10.1021/acs.jproteome.6b00156 27052453

[B13] Kauppinen A. Suuronen T. Ojala J. Kaarniranta K. Salminen A. (2013). Antagonistic crosstalk between NF-κB and SIRT1 in the regulation of inflammation and metabolic disorders. Cell Signal 25, 1939–1948. 10.1016/j.cellsig.2013.06.007 23770291

[B14] Kern F. Fehlmann T. Solomon J. Schwed L. Grammes N. Backes C. (2020). miEAA 2.0: integrating multi-species microRNA enrichment analysis and workflow management systems. Nucleic Acids Res. 48, W521–W528. 10.1093/nar/gkaa309 32374865 PMC7319446

[B15] Landi C. Bargagli E. Carleo A. Bianchi L. Gagliardi A. Prasse A. (2014). A system biology study of BALF from patients affected by idiopathic pulmonary fibrosis (IPF) and healthy controls. Proteomics Clin. Appl. 8, 932–950. 10.1002/prca.201400001 25169739

[B16] Ley B. Brown K. K. Collard H. R. (2014). Molecular biomarkers in idiopathic pulmonary fibrosis. Am. J. Physiol. Lung Cell Mol. Physiol. 307, L681–L691. 10.1152/ajplung.00014.2014 25260757 PMC4280147

[B17] Ley B. Collard H. R. King T. E. JR. (2011). Clinical course and prediction of survival in idiopathic pulmonary fibrosis. Am. J. Respir. Crit. Care Med. 183, 431–440. 10.1164/rccm.201006-0894CI 20935110

[B18] Liberzon A. Subramanian A. Pinchback R. Thorvaldsdottir H. Tamayo P. Mesirov J. P. (2011). Molecular signatures database (MSigDB) 3.0. Bioinformatics 27, 1739–1740. 10.1093/bioinformatics/btr260 21546393 PMC3106198

[B19] Liu J. Wu Z. Liu Y. Zhan Z. Yang L. Wang C. (2022). ROS-responsive liposomes as an inhaled drug delivery nanoplatform for idiopathic pulmonary fibrosis treatment via Nrf2 signaling. J. Nanobiotechnology 20, 213. 10.1186/s12951-022-01435-4 35524280 PMC9074278

[B20] Liu X. Ostrom R. S. Insel P. A. (2004). cAMP-elevating agents and adenylyl cyclase overexpression promote an antifibrotic phenotype in pulmonary fibroblasts. Am. J. Physiol. Cell Physiol. 286, C1089–C1099. 10.1152/ajpcell.00461.2003 15075208

[B21] Lubos E. Loscalzo J. Handy D. E. (2011). Glutathione peroxidase-1 in health and disease: From molecular mechanisms to therapeutic opportunities. Antioxid. Redox Signal 15, 1957–1997. 10.1089/ars.2010.3586 21087145 PMC3159114

[B22] Maher T. M. Bendstrup E. Dron L. Langley J. Smith G. Khalid J. M. 2021. Global incidence and prevalence of idiopathic pulmonary fibrosis. Respir. Res., 22, 197, 10.1186/s12931-021-01791-z 34233665 PMC8261998

[B23] Mamazhakypov A. Schermuly R. T. Schaefer L. Wygrecka M. (2019). Lipids - two sides of the same coin in lung fibrosis. Cell Signal 60, 65–80. 10.1016/j.cellsig.2019.04.007 30998969

[B24] Martin J. L. Begun J. Mcleish M. J. Caine J. M. Grunewald G. L. (2001). Getting the adrenaline going: Crystal structure of the adrenaline-synthesizing enzyme PNMT. Structure 9, 977–985. 10.1016/s0969-2126(01)00662-1 11591352

[B25] Moeller A. Ask K. Warburton D. Gauldie J. Kolb M. 2008. The bleomycin animal model: A useful tool to investigate treatment options for idiopathic pulmonary fibrosis? Int. J. Biochem. Cell Biol., 40, 362, 10.1016/j.biocel.2007.08.011 17936056 PMC2323681

[B26] Newman A. M. Liu C. L. Green M. R. Gentles A. J. Feng W. Xu Y. (2015). Robust enumeration of cell subsets from tissue expression profiles. Nat. Methods 12, 453–457. 10.1038/nmeth.3337 25822800 PMC4739640

[B27] Noble P. W. Barkauskas C. E. Jiang D. (2012). Pulmonary fibrosis: Patterns and perpetrators. J. Clin. Invest. 122, 2756–2762. 10.1172/JCI60323 22850886 PMC3408732

[B28] Pernicova I. Korbonits M. (2014). Metformin--mode of action and clinical implications for diabetes and cancer. Nat. Rev. Endocrinol. 10, 143–156. 10.1038/nrendo.2013.256 24393785

[B29] Raghu G. Remy-Jardin M. Myers J. L. Richeldi L. Ryerson C. J. Lederer D. J. (2018). Diagnosis of idiopathic pulmonary fibrosis. An official ATS/ERS/JRS/ALAT clinical practice guideline. Am. J. Respir. Crit. Care Med., 198, e44, 10.1164/rccm.201807-1255ST 30168753

[B30] Richter K. Kietzmann T. (2016). Reactive oxygen species and fibrosis: Further evidence of a significant liaison. Cell Tissue Res. 365, 591–605. 10.1007/s00441-016-2445-3 27345301 PMC5010605

[B31] Saito S. Alkhatib A. Kolls J. K. Kondoh Y. Lasky J. A. (2019). Pharmacotherapy and adjunctive treatment for idiopathic pulmonary fibrosis (IPF). J. Thorac. Dis. 11, S1740–S1754. 10.21037/jtd.2019.04.62 31632751 PMC6783717

[B32] Sasaki T. Kotera J. Yuasa K. Omori K. (2000). Identification of human PDE7B, a cAMP-specific phosphodiesterase. Biochem. Biophys. Res. Commun. 271, 575–583. 10.1006/bbrc.2000.2661 10814504

[B33] Shannon P. Markiel A. Ozier O. Baliga N. S. Wang J. T. Ramage D. (2003). Cytoscape: A software environment for integrated models of biomolecular interaction networks. Genome Res. 13, 2498–2504. 10.1101/gr.1239303 14597658 PMC403769

[B34] Shen W. Song Z. Zhong X. Huang M. Shen D. Gao P. 2022. Sangerbox: A comprehensive, interaction-friendly clinical bioinformatics analysis platform. iMeta, 1, e36, 10.1002/imt2.36 PMC10989974 38868713

[B35] Shenderov K. Collins S. L. Powell J. D. Horton M. R. (2021). Immune dysregulation as a driver of idiopathic pulmonary fibrosis. J. Clin. Invest. 131, e143226. 10.1172/JCI143226 33463535 PMC7810481

[B36] Sherman B. T. Hao M. Qiu J. Jiao X. Baseler M. W. Lane H. C. (2022). David: A web server for functional enrichment analysis and functional annotation of gene lists (2021 update). Nucleic Acids Res. 50, W216–W221. 10.1093/nar/gkac194 35325185 PMC9252805

[B37] Sriram K. Insel M. B. Insel P. A. (2021). Inhaled beta2 adrenergic agonists and other cAMP-elevating agents: Therapeutics for alveolar injury and acute respiratory disease syndrome? Pharmacol. Rev. 73, 488–526. 10.1124/pharmrev.121.000356 34795026

[B38] Sticht C. De La Torre C. Parveen A. Gretz N. (2018). miRWalk: An online resource for prediction of microRNA binding sites. PLoS One 13, e0206239. 10.1371/journal.pone.0206239 30335862 PMC6193719

[B39] Torphy T. J. (1994). Beta-adrenoceptors, cAMP and airway smooth muscle relaxation: Challenges to the dogma. Trends Pharmacol. Sci. 15, 370–374. 10.1016/0165-6147(94)90157-0 7809952

[B40] Vasse G. F. Nizamoglu M. Heijink I. H. Schleputz M. Van Rijn P. Thomas M. J. (2021). Macrophage-stroma interactions in fibrosis: Biochemical, biophysical, and cellular perspectives. J. Pathol. 254, 344–357. 10.1002/path.5632 33506963 PMC8252758

[B41] Wickham H. (2016). “Data analysis,” in ggplot2: Elegant graphics for data analysis. Editor WICKHAM H. (Cham: Springer International Publishing).

[B42] Wynn T. A. Vannella K. M. (2016). Macrophages in tissue repair, regeneration, and fibrosis. Immunity 44, 450–462. 10.1016/j.immuni.2016.02.015 26982353 PMC4794754

[B43] Xie N. Tan Z. Banerjee S. Cui H. Ge J. Liu R. M. (2015). Glycolytic reprogramming in myofibroblast differentiation and lung fibrosis. Am. J. Respir. Crit. Care Med., 192, 1462, 10.1164/rccm.201504-0780OC 26284610 PMC4731722

