# Supplementary material for: Identification and validation of metabolism-related hub genes in idiopathic pulmonary fibrosis
Source: Front Genet. 2023 Feb 27;14:1058582. doi: 10.3389/fgene.2023.1058582 (PMC10010493; doi:10.3389/fgene.2023.1058582)
Supplement: Supplementary file 2 [file Table2.pdf]

Supplementary Table S2 Shapiro-Wilk test for the amount of the immune cells.

Immune cells	IPF		Control	
	W	P-value	W	P-value
Mast.cells.activated	0.065878	**4.22E-24**	NA	NA
T.cells.CD4.naive	0.161265	**4.75E-23**	0.21624	**7.21E-15**
Dendritic.cells.resting	0.324659	**5.20E-21**	0.125428	**1.13E-15**
Eosinophils	0.329135	**5.99E-21**	0.612535	**3.00E-10**
T.cells.regulatory..Tregs.	0.355861	**1.41E-20**	NA	NA
T.cells.CD4.memory.activated	0.426318	**1.53E-19**	0.421574	**9.29E-13**
T.cells.follicular.helper	0.429868	**1.74E-19**	0.125428	**1.13E-15**
Macrophages.M1	0.515974	**4.51E-18**	0.363447	**2.10E-13**
Neutrophils	0.691549	**1.77E-14**	0.950228	**0.03486**
B.cells.memory	0.692232	**1.84E-14**	0.496305	**7.40E-12**
NK.cells.resting	0.817635	**7.77E-11**	0.979403	0.527049
Dendritic.cells.activated	0.885355	**4.15E-08**	0.906907	**0.000819**
NK.cells.activated	0.940392	**4.89E-05**	0.909635	**0.001016**
B.cells.naive	0.947961	**0.000164**	0.952336	**0.042585**
T.cells.CD8	0.948774	**0.000188**	0.966794	0.170892
Plasma.cells	0.965044	**0.00346**	0.975692	0.387665
Macrophages.M0	0.969486	**0.008279**	0.962881	0.117381
Mast.cells.resting	0.976701	**0.03655**	0.983912	0.724014
T.cells.CD4.memory.resting	0.97773	**0.045417**	0.958141	0.074299
Macrophages.M2	0.991855	0.712742	0.977269	0.44341
Monocytes	0.994206	0.9073	0.901895	**0.000555**

Supplementary Table S3 Shapiro-Wilk test for the relative mRNA expression of key DEMRGs in the GSE53845 dataset

	Group	W	P-value
ENPP3	IPF	0.96887	0.3313
	con	0.93704	0.5822
PDE7B	IPF	0.94224	**0.04116**
	con	0.97234	0.9157
ENTPD1	IPF	0.98116	0.7326
	con	0.98862	0.9927
PNMT	IPF	0.77883	**2.52E-06**
	con	0.9355	0.5674
GPX3	IPF	0.97377	0.4697
	con	0.95041	0.7154
POLR3H	IPF	0.8915	**0.001092**
	con	0.88805	0.2244

Supplementary Table S4 Shapiro-Wilk test for the relative mRNA expression of key DEMRGs in the GSE213001 dataset

	Group	W	P-value
ENPP3	IPF	0.91018	**0.00025**
	con	0.98889	0.9549
PDE7B	IPF	0.98556	0.6797
	con	0.99153	0.9883
ENTPD1	IPF	0.93952	**0.004306**
	con	0.98308	0.7903
PNMT	IPF	0.94563	**8.26E-03**
	con	0.93118	**0.01581**
GPX3	IPF	0.98773	0.7931
	con	0.97724	0.5723
POLR3H	IPF	0.95075	**0.0145**
	con	0.97304	0.4309

Supplementary Table S5 Shapiro-Wilk test for the relative mRNA expression of key genes examined by qPCR in BLM group

	W	P-value
ENPP3	0.96328	0.8446
PDE7B	0.94941	0.7355
ENTPD1	0.85401	0.1695
PNMT	0.89529	0.3468
GPX3	0.85656	0.1777
POLR3H	0.97182	0.9044
